# Repurposing Eltrombopag for Multidrug Resistant *Staphylococcus aureus* Infections

**DOI:** 10.3390/antibiotics10111372

**Published:** 2021-11-09

**Authors:** Hyunjung Lee, Jaehoan Lee, Juchan Hwang, Sinyoung Park, Namyoul Kim, Kideok Kim, Honggun Lee, David Shum, Soojin Jang

**Affiliations:** 1 Antimicrobial Resistance Laboratory, Institut Pasteur Korea, Seongnam-si 13488, Korea; hyunjung.lee@ip-korea.org (H.L.); Jellyfocus@outlook.com (J.L.); juchan.hwang@ip-korea.org (J.H.); 2 Animal Facility Team, Institut Pasteur Korea, Seongnam-si 13488, Korea; psy6600@daum.net; 3 Screening Discovery Platform, Institut Pasteur Korea, Seongnam-si 13488, Korea; skaduf@hanmail.net (N.K.); kideok.kim@ip-korea.org (K.K.); honggun.lee@ip-korea.org (H.L.); david.shum@ip-korea.org (D.S.)

**Keywords:** *Staphylococcus aureus*, MRSA, drug repurposing, eltrombopag, in vivo efficacy

## Abstract

The continuous rise of antimicrobial resistance urgently demands new therapeutic agents for human health. Drug repurposing is an attractive strategy that could significantly save time delivering new antibiotics to clinics. We screened 182 US Food and Drug Administration (FDA)-approved drugs to identify potential antibiotic candidates against *Staphylococcus aureus*, a major pathogenic bacterium. This screening revealed the significant antibacterial activity of three small molecule drugs against *S. aureus*: (1) LDK378 (Ceritinib), an anaplastic lymphoma kinase (ALK) inhibitor for the treatment of lung cancer, (2) dronedarone HCl, an antiarrhythmic drug for the treatment of atrial fibrillation, and (3) eltrombopag, a thrombopoietin receptor agonist for the treatment of thrombocytopenia. Among these, eltrombopag showed the highest potency against not only a drug-sensitive *S. aureus* strain but also 55 clinical isolates including 35 methicillin-resistant *S. aureus* (Minimum inhibitory concentration, MIC, to inhibit 50% growth [MIC_50_] = 1.4–3.2 mg/L). Furthermore, we showed that eltrombopag inhibited bacterial growth in a cell infection model and reduced bacterial loads in infected mice, demonstrating its potential as a new antibiotic agent against *S. aureus* that can overcome current antibiotic resistance.

## 1. Introduction

Antibiotic resistance is one of the greatest threats to global public health, which could cause the next pandemic. Development of effective therapeutic agents is vital to prepare for a battle against antibiotic resistant infections. *S. aureus* is a leading cause of bacterial infections worldwide and remains a major public health concern due to the emergence and rapid spread of drug-resistant strains, such as methicillin-resistant *S. aureus* (MRSA) and the vancomycin-resistant strain that was reported in the United States in 2002 [1]. The World Health Organization (WHO) has listed *Staphylococcus aureus* as a priority pathogen for the urgent development of new antibiotics [2]. However, the development of new antibiotics for clinical usage can take more than 10 years. Alternatively, repositioning currently approved drugs that may have antimicrobial [3] or antiviral [4] activities could shorten the development time substantially.

In this study, we assessed 182 Food and Drug Administration (FDA)-approved drugs for their potential antimicrobial activity against *S. aureus*. Three drugs demonstrated promising results—Ceritinib, dronedarone HCl, and eltrombopag. Among them, eltrombopag, a non-peptide agonist of the thrombopoietin receptor (TpoR) initially approved for the treatment of thrombocytopenia, showed the greatest efficacy against *S. aureus* including 55 clinical isolates. We confirmed that not only in vitro but also ex vivo and in vivo efficacy of eltrombopag demonstrating that it is a good drug candidate for treatment of multi-drug resistant *S. aureus* infections.

## 2. Results and Discussion

### 2.1. Primary Screening

**Figure 1:**
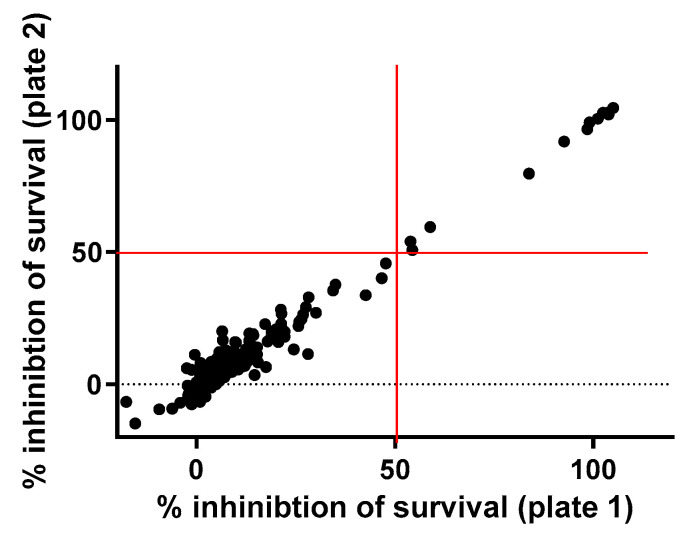
Primary screen of the drug library. A total of 182 FDA-approved compounds were tested against *S. aureus* at 10 µM. A total of 12 drugs inhibited the growth of bacteria by more than 50%.

A total of 182 FDA-approved drugs were selected from the compound collection at the Institut Pasteur Korea (IPK) and screened for antibacterial activities against *S. aureus* ATCC25923 at 10 µM. The primary screening identified 12 compounds that inhibited bacterial growth by more than 50% (Figure 1, Table 1), including 8 known to be antimicrobial molecules and 4 miscellaneous repositionable candidates: dronedarone HCl, ceritinib, eltrombopag and ivacaftor. In this study ivacaftor, a drug for cystic fibrosis, was excluded for further investigation because antibacterial activity of ivacaftor has been reported against *S. aureus* [3]. The primary screening successfully identified all known antibiotics among the 182 FDA-approved drugs that were tested, validating the high fidelity of the antibacterial assay.

### 2.2. Dose–Response Assay of Selected Repositionable Candidates

**Figure 2:**
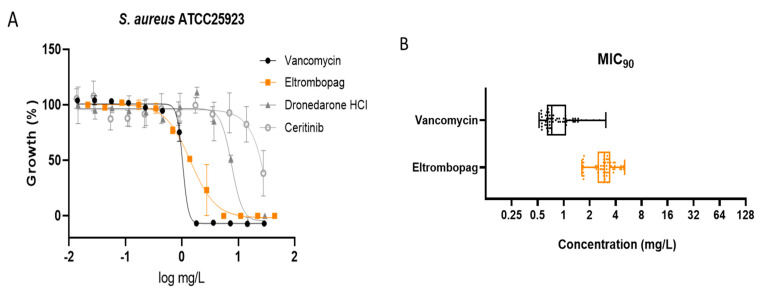
Antimicrobial activity of eltrombopag compound. (**A**) Dose-response curves for dronedarone HCl, ceritinib, eltrombopag and vancomycin (0.014–30 mg/L, 0.014–28 mg/L, 0.022–44.25 mg/L and 0.014–29 mg/L) against *S. aureus* ATCC25923. The data represent the mean ± SD (*n* = 3 per group). (**B**) Distribution of eltrombopag MIC_90_ against MDR #1−5, MSSA #1−20 and MRSA #1−30 strains.

We tested the three selected repositionable drug candidates against *S. aureus* ATCC25923 at concentrations ranging from 0.014 mg/L to 44.25 mg/L (using 2-fold dilutions) to confirm their antibacterial activities and determine their potencies with minimum inhibitory concentration (MIC) values (Figure 2A).

Dronedarone HCl is a Class III antiarrhythmic drug that works to restore the normal sinus rhythm in patients with paroxysmal or persistent atrial fibrillation. Amiodarone, the parent drug of dronedarone HCl, has shown antibacterial activity and has been suggested as a catheter lock to prevent bacterial infection [5]. Similarly, we found that the concentration that inhibits 50% of the bacterial growth (MIC_50_) of dronedarone HCl is 7.6 ± 0.7 mg/L.

Several studies describe the antibacterial activities of anticancer drugs. Sorafenib, a human multi-kinase inhibitor approved as an anticancer drug, is active against *S. aureus* [6]. A developed analog of sorafenib, PK150, showed improved antibacterial activity [7]. Our screening also identified the antibacterial activity of ceritinib, an anticancer drug that inhibits anaplastic lymphoma kinase (ALK) [8]. The MIC_50_ of ceritinib in our study was 23.7 ± 5.3 mg/L against *S. aureus*. Although its potency is not sufficient to serve as an independent antibiotic, ceritinib could be a seed molecule for further development, similar to sorafenib.

Eltrombopag was originally developed to treat certain conditions that lead to thrombocytopenia. Eltrombopag is a small molecule agonist of the platelet thrombopoietin receptor (TpoR) that binds to and stimulates TpoR, increasing the production of blood platelets [9]. We found that eltrombopag has the highest potency among the three identified repositioning candidates (MIC_50_ = 1.5 ± 0.1 mg/L). Since eltrombopag is an orally bioavailable drug, which is a beneficial feature for antibiotics, we performed subsequent investigations to demonstrate and validate the antibacterial activity of eltrombopag against clinically isolated and multidrug resistant *S. aureus*.

### 2.3. Confirmation of Eltrombopag Activity against S. aureus Clinical Isolates and Other Bacterial Species

We performed the antibacterial activity test of eltrombopag against fifty-five clinically isolates of *S. aureus* including 30 MRSA strains, some of which were resistant to multiple antibiotics such as fluoroquinolones (levofloxacin), tetracycline, erythromycin, beta-lactams (penicillin and methicillin), and second- and third-generation cephalosporins (cefoxitin, cefoperazone, and ceftriazone) with and without a beta-lactamase inhibitor (sulbactam) (Supplementary Table S1). Eltrombopag was active against all fifty-five clinical isolates (*S. aureus* MDR#1−5, MSSA#1−20, and MRSA#1−30) within the range of MIC_90_ between 1.6 and 5.1 mg/L with a median value of 3.0 mg/L, which was similar to observed activity against the drug-sensitive *S. aureus*. The result suggested that eltrombopag likely acts through an antibacterial mechanism distinct from those of current antibiotics (Figure 2B and Supplementary Table S2). We next determined an antibacterial spectrum for eltrombopag against additional Gram-positive bacteria (*Streptococcus pneumoniae*) and Gram-negative bacteria (*Pseudomonas aeruginosa*, *Acinetobacter baumannii*, and *Klebsiella pneumoniae*). We found that eltrombopag efficiently inhibited pneumococcal growth (MIC_50_ = 0.3 mg/L) but showed no activity against the three Gram-negative bacteria (Supplementary Table S2). These results suggested that eltrombopag likely has the activity against Gram-positive bacteria but not Gram-negative bacteria, which might cause less collateral damages and resistance in the resident microbiota compared to broad spectrum antibiotics [10].

### 2.4. Eltrombopag Inhibits S. aureus Growth in a Cell Line Infection Model

**Figure 3:**
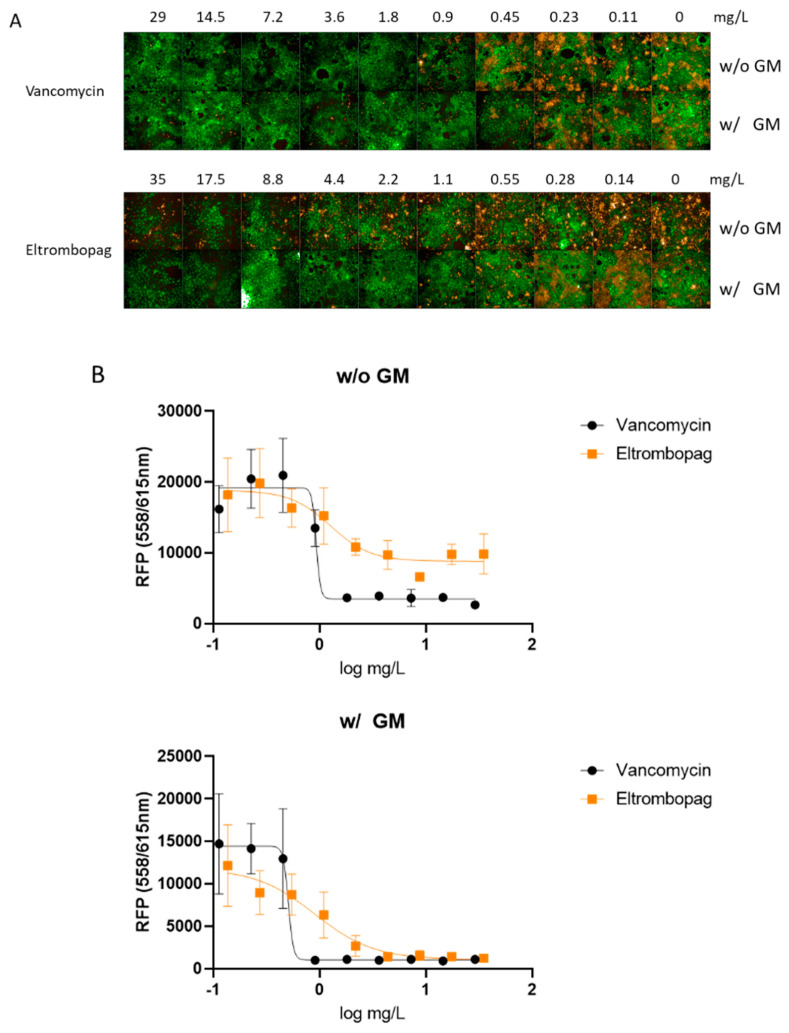
Ex vivo activity of eltrombopag in the Caco-2 infection model. (**A**) The confocal microscope images show Caco-2 cells (green) and *S. aureus* infection (orange) at each antibiotic concentration. (**A**) *S. aureus* infected Caco-2 cells were treated with vancomycin (0.11–29 mg/L) or eltrombopag (0.14–35 mg/L). Each experimental group was treated with or without gentamicin. (**B**) Dose−response curves for vancomycin (0.11–29 mg/L) and eltrombopag (0.14–35 mg/L), with or without gentamicin. The data represent the mean ± SD (*n* = 3 per group).

Previous studies suggested that the intestinal colonization of *S. aureus* may cause antibiotic-associated enterocolitis and serve as a reservoir for staphylococcal infections [11,12,13]. It has been reported that *S. aureus* coexists with vancomycin-resistant Enterococcus in the intestinal tract of more than 50% of American patients, raising the concern of emerging vancomycin-resistant *S. aureus* [14]. Therefore, we set up an infection model using the human intestinal Caco-2 cells to assess whether eltrombopag could eradicate colonized *S. aureus* from the intestines. Caco-2 cells were infected with *S. aureus* expressing red fluorescence protein (RFP) and treated with eltrombopag. Antibacterial activity specifically against cell invaded bacteria was determined after killing and removing extracellular bacteria by gentamicin treatment prior to eltrombopag. After a 24-h incubation, we measured eltrombopag activity by detecting *S. aureus* growth based on the RFP signal and measured Caco-2 cell viability using the CellTracker Green CMFDA Dye. We found that *S. aureus* growth decreased by eltrombopag in both conditions with (MIC_50_ = 1.2 ± 0.6 mg/L) and without (MIC_50_ = 1.5 ± 0.2 mg/L) gentamicin treatment similar to vancomycin (Figure 3A,B). Cytotoxicity of eltrombopag was tested with Caco-2 and HepG2 cell lines. The 50% inhibitory concentration (IC_50_) of eltrombopag for Caco-2 and HepG2 was 202.8 mg/L and 170 mg/L, respectively. The selectively index (SI) of eltrombopag was 63 for Caco-2 and 53 for HepG2 (data not shown). These results suggest that eltrombopag can be used against intestinally colonized *S. aureus* with a minimum cytotoxicity.

### 2.5. In Vivo Efficacy of Eltrombopag in a Mouse Infection Model

**Figure 4:**
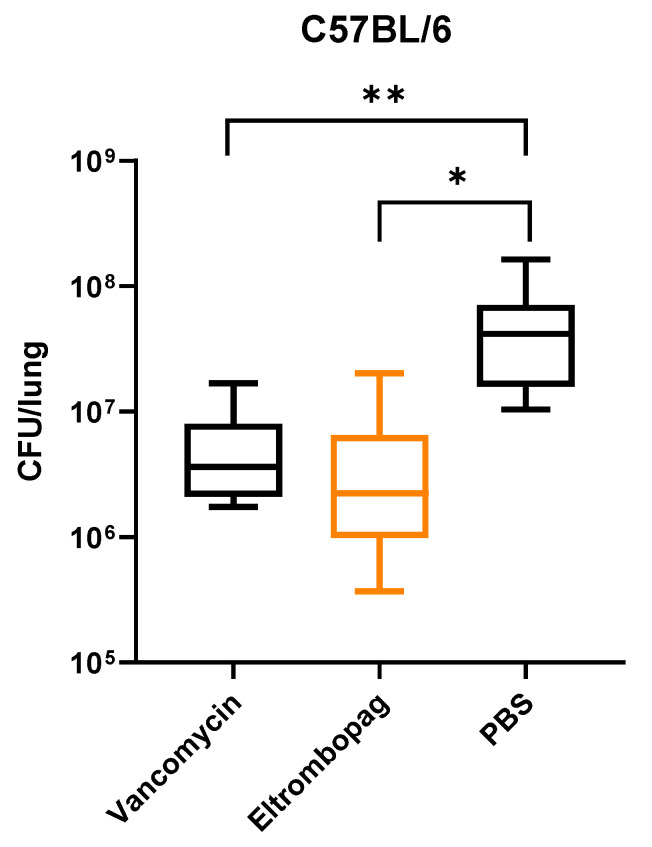
In vivo activity of eltrombopag in mouse nasal infection model. C57BL/C mice were infected by intranasal injection of *S. aureus*. After infection, mice were treated by intraperitoneal (IP) injection of either vancomycin or eltrombopag. Control mice received IP injection of PBS. CFU analysis showed relatively low bacterial loads in the lungs of mice treated with vancomycin (5.5 × 10^6^ CFU/lung) or eltrombopag (5.0 × 10^6^ CFU/lung) compared with PBS (5.2 × 10^7^ CFU/lung) controls. The data represent the mean to max values (*n*=10 per group). Statistical significance was determined by *t*-test (*, *p* = 0.007 and **, *p* = 0.007).

As eltrombopag exhibited potency in our in vitro assay and cell infection model, we then wanted to verify its in vivo efficacy in a mouse infection model. We nasally infected male C57BL/6 mice with 5 × 10^8^ CFU of *S. aureus*. At 30 min post-infection, we treated mice with either 50 mg/kg of vancomycin or 17.6 mg/kg of eltrombopag by intraperitoneal injection once a day for 2 days. Treatment with eltrombopag (5.0 × 10^6^ CFU/lung) significantly reduced mean bacterial counts in the nasal infection model compared with control PBS (5.2 × 10^7^ CFU/lung) mice (*p* < 0.05; Figure 4). We obtained similar results with vancomycin (5.5 × 10^6^ CFU/lung) treatment, demonstrating that eltrombopag exhibits great potential for repurposing as an antibacterial therapeutic agent, especially against *S. aureus*.

### 2.6. Elucidating the Antibacterial Mechanism of Eltrombopag

**Figure 5:**
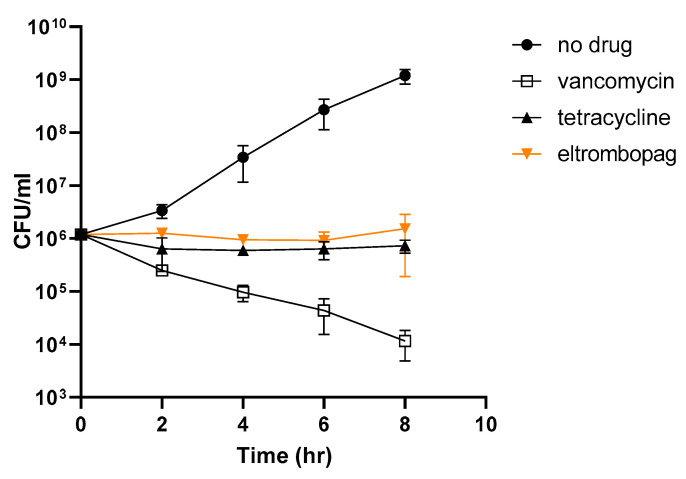
Bacterial killing effect of eltrombopag. Time-dependent killing of exponentially growing *S. aureus* wild-type (wt), with or without antibiotics (vancomycin and tetracycline) or eltrombopag. Antibiotics were tested at 2× the MIC_50_ value. Data represent the mean ± SD (*n* = 2 per group). Each experiment was tested three times using biologically independent samples.

To understand the mechanism of antibacterial activity by eltrombopag, we performed a time-kill kinetics assay using tetracycline and vancomycin as bacteriostatic and bactericidal antibiotic controls, respectively. When we exposed bacteria (*S. aureus* ATCC25923) to each drug, vancomycin reduced the number of viable bacteria over time, whereas tetracycline did not, as expected for a bacteriostatic antibiotic (Figure 5). Similar to tetracycline, eltrombopag did not reduce the number of viable bacteria (Figure 5), suggesting that eltrombopag is a bacteriostatic agent.

**Figure 6:**
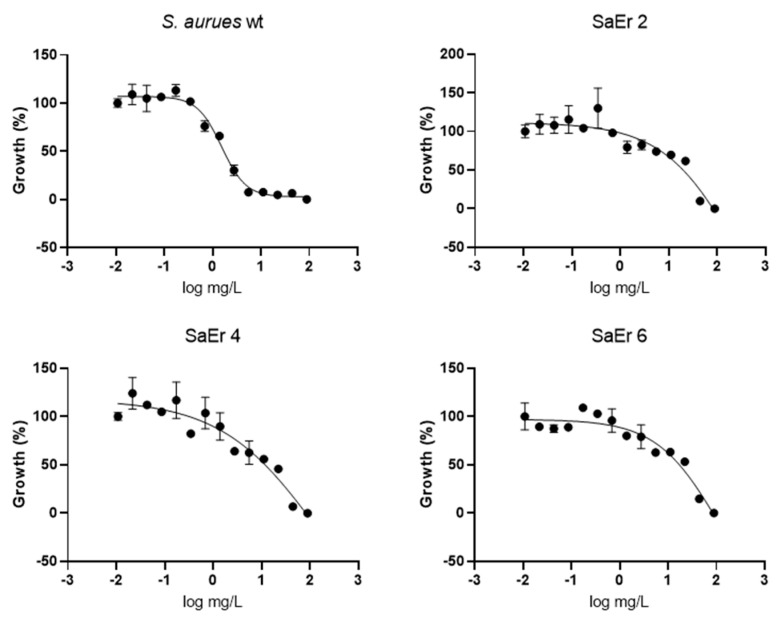
Antimicrobial activity of eltrombopag compound. Dose–response curve of eltrombopag (0.02−88 mg/L) against *S. aureus* ATCC25923 and eltrombopag resistant strains.

The efficacy of eltrombopag as a therapeutic drug for thrombocytopenia is based on its activation of TpoR by selective binding to the trans-membrane domain of the TpoR [15]. Because *S. aureus* does not possess any known homologs of TpoR, how eltrombopag inhibits bacterial growth remains unclear. To elucidate the antibacterial mechanism of eltrombopag, we attempted to obtain spontaneous resistant mutants by pre-exposing the bacteria at various concentrations of eltrombopag, which resulted in 5.5 × 10^−9^ of a spontaneous resistant mutation frequency. Three selected resistant clones showed at least a 5–10-fold reduction of eltrombopag susceptibility (Figure 6 and Supplementary Table S2). The whole genome sequencing analysis revealed that all three sequenced resistant clones possess mutations in the same five genes: *ydeL*, (known as *pmtR*) a toxin-mediated transcriptional regulator, *walR*, a DNA-binding response regulator of WalRK two-component system, *yjbH* (known as *spxH*), an adapter protein involved in Spx degradation, *lytE*, a D, L-endopeptidase, and *yokF*, an endonuclease (Table 2). Point mutations were found in *ydeL* (P37L) and *walR* (E11G or D83G), two transcriptional regulators, which potentially alter their gene regulatory function affecting transcriptional levels of many genes in their regulons as we observed inductions of *pmtA* and *pmtD* in the YdeL regulon (Supplementary Figure S1). Base insertion or deletion mutations occurred in the other three genes including *yjbH*, *lytE*, and *yokF*, which likely cause complete loss of their functions resulted from protein truncation. Since the abnormal presence of autolysins and endonucleases such as LytE and YokF can cause bacterial death and these enzymes were found to be impaired in the eltrombopag resistant clones, it implies that eltrombopag-mediated bacterial growth inhibition might be associated with the activity of LytE and YokF [16,17]. The mutation of *yjbH* found in the resistant mutants likely results in decreased degradation of Spx, a stress response gene regulator suggesting the pivotal role of the stress response for the bacterial resistance of eltrombopag [18].

Taken together, the genomic analysis of the resistant clones suggested that the inactivation of LytE and YokF as well as the orchestration of gene expression in the YdeL, WalR, and Spx regulons contributed to bacterial survival in the presence of eltrombopag. Further investigations remain necessary to elucidate the molecular antibacterial mechanism of eltrombopag.

## 3. Materials and Methods

### 3.1. Strains and Culture Conditions

*Staphylococcus aureus* ATCC25923, a pan-drug susceptible strain, *Streptococcus pneumoniae* ATCC49169, *Streptococcus pneumoniae* ATCC700904, *Acinetobacter baumannii* ATCC19606, *Klebsiella pneumoniae* ATCC13883 were purchased from the American Type Culture Collection (ATCC). *Pseudomonas aeruginosa* and 5 strains of multidrug resistant *S. aureus* were obtained from Yonsei University (Supplementary Table S1). Fifty of clinically isolates of *S. aureus* including 30 of methicillin-resistant *S. aureus* (MRSA) were obtained from Seoul National University Bundang Hospital (Supplementary Table S2). *S. aureus* strains were grown in Mueller-Hinton broth (MHB) (Difco) at 37 °C. *Pseudomonas aeruginosa*, *Acinetobacter baumannii*, and *Klebsiella pneumoniae* strains were grown in Luria-Bertani (LB) broth at 37 °C. Bacterial strain stocks were stored in vials with 25% glycerol at −80 °C. For each experiment, overnight cultures were prepared by inoculating stock in 10 mL of either MHB or LB broth medium and incubating at 37 °C in a shaking incubator (180 rpm). For the preliminary screen and dose–response experiments, 100 µL of overnight culture was inoculated into 10 mL fresh MHB or LB broth and incubated for 3 h at 37 °C and 180 rpm.

### 3.2. Primary Screening

We performed a pilot screen of 182 FDA-approved compounds currently housed in a collection at the Institut Pasteur Korea (Seongnam-si, South Korea). We diluted frozen stock of *S. aureus* strain 1:100 in MHB and incubated cultures overnight at 37 °C and 180 rpm. The overnight culture was subsequently diluted 1:100 in fresh MHB and incubated for 3 h at 37 °C, 180 rpm to 0.5 of OD_600_. We then added 40 µL of *S. aureus* in each well of 348 well microplates where each compound had been dispensed at 10 µM in 10 µL of 0.5% dimethyl sulfoxide [DMSO]. After incubation of the plates at 37 °C overnight, the OD was obtained using a Victor III (PerkinElmer, Waltham, MA, USA).

### 3.3. Dose–Response of Selected Repositionable Candidates

We conducted antibiotic susceptibility testing according to modified Clinical and Laboratory Standards Institute (CLSI) laboratory guidelines for broth microdilution assays [19]. We inoculated bacterial cultures (*S. aureus*, *P. aeruginosa*, *A. baumannii*, and *K. pneumoniae*) in MHB or LB until cultures and prepared bacterial cells OD_600_ of 0.05. DRC plates were prepared by adding 45 µL bacterial suspension to each well where 5 µL of each compound had been dispensed for 13 points of two-fold serial dilutions starting at 22 mg/L. After incubation of the plates at 37 °C for 18–24 h with 5% CO_2_, we detected *S. aureus* growth by measuring the OD_600_ using a Victor III spectrometer.

### 3.4. Effect of Eltrombopag on S. aureus Infection of Human Caco-2 Cells

We used the human intestinal cell line Caco-2 in ex vivo antimicrobial activity tests of eltrombopag and vancomycin as a positive control drug. We cultured Caco-2 cells in minimum essential media (MEM) with 10% fetal bovine serum (FBS) and 1× non-essential amino acids in a 96-well microplate at 37 °C and 5% CO_2_. After Caco-2 cells reached full confluence, we grew the cells and changed media every 2 days for 5 more days to obtain differentiated cells in 384-well plates, as described in previous reports, with some modifications (Materials and Methods) [20,21,22]. We cultured *S. aureus* (ATCC25923 containing a pHC48 encoding dsRedExpress) in MHB with 10 µg/mL of chloramphenicol and harvested bacteria cells [23]. After washing the bacterial cells with phosphate-buffered saline (PBS) and suspending them in MEM, we infected Caco-2 cells with *S. aureus* (1 × 10^7^ CFU) and incubated them for 2.5 h. We then treated cells with gentamicin (50 µg/mL of gentamicin in MEM) and incubated plates for 1 h. We washed samples two times with PBS, added MEM containing diluted compounds, and incubated plates at 37 °C in 5% CO_2_ for 24 h. We added CellTrackerTM Green 5-Chloromethylfluorescein diacetate (CMFDA) Dye (Thermo Fisher Scientific, Waltham, MA, USA) to stain live Caco-2 cell, incubated the plates for 1 h, and imaged the plates using Operetta CLS (PerkinElmer, Waltham, MA, USA) to obtain images. To detect the dsRedExpress fluorescent protein (RFP) signal, we used an Ensight multimode plate reader (PerkinElmer, Waltham, MA, USA).

### 3.5. Cytotoxicity Test of Eltrombopag

We used the human liver cell line HepG2 and intestinal cell line Caco-2 in the cytotoxicity test of eltrombopag. We cultured HepG2 and Caco-2 cells in DMEM with 10% fetal bovine serum (FBS) in a 96-well microplate at 37 °C and 5% CO_2_. HepG2 and Caco-2 cells were seeded at a final density of 5 × 10^4^ cells/well and DMEM containing diluted compounds was added, and the plates were incubated at 37 °C in 5% CO_2_ for 24 h. We added resazurin (final concentration, 0.1 mg/mL) to each well and, incubated the plates at 37 °C in 5% CO_2_ for 2 h. To detect the fluorescence at 530 nm excitation and 590 nm emission, we used an Ensight multimode plate reader (PerkinElmer, Waltham, MA, USA). Selective activities of the compounds were calculated as follows:

Selectivity index (SI) = IC_50_ cell lines/ IC_50_ inhibition concentration to bacterial growth

### 3.6. Mouse Infection Model

7-week-old C57BL/6 male mice (Orient Bio, Inc., Seongnam-si, South Korea) weighing 20–22 g were used to test the in vivo antimicrobial activity of eltrombopag against *S. aureus* ATCC25923 (protocol approved by The Animal Care and Ethics Committee of IPK, Approval numbers IPK-19006 and IPK-20009). We injected *S. aureus* (5 × 10^8^ CFU suspended in 40 µL PBS) into the nasal cavities of anesthetized mice (*n* = 10 per group). At 30 min post-infection, eltrombopag (17.6 mg/kg body weight) and vancomycin (50 mg/kg body weight) were administrated by intraperitoneal injection two times 24 h apart. Due to the lower solubility of eltrombopag, 17.6 mg/kg was the highest solubilized concentration we tested in this study. A control group was injected with PBS in the same volume and frequency as mice receiving treatment. After 24 h, we sacrificed the mice and collected lung tissue. We homogenized the collected lung tissue in 1 mL PBS, serially diluted the homogenate, and plated dilutions on LB plates. We incubated plates for 18–24 h at 37 °C and counted bacterial colonies to determine the CFU.

### 3.7. Kinetics of Time-Dependent Bacterial Killing

We diluted overnight cultures of *S. aureus* ATCC25923 to 10^6^ CFU/mL in 10 mL MHB supplemented with an MIC of antimicrobials (vancomycin, tetracycline, and eltrombopag) and incubated cultures at 37 °C with shaking at 180 rpm. We collected bacterial cells (0.1 mL) from the culture at different incubation times, serially diluted collected cells with PBS, and plated cells on MH agar plates. We counted surviving colonies after incubating plates for 24 h at 37 °C. We repeated each experiment three times in duplicate.

### 3.8. Eltrombopag-Resistant Mutant Selection

To generate an eltrombopag-resistant mutant of *S. aureus*, we cultured bacterial cells in MHB at 37 °C. We harvested cells at the early stationary phase of *S. aureus* growth and suspended the cells in MHB at 2 × 10^9^ CFU/100 µL. We plated *S. aureus* on MH agar plates with various concentrations of eltrombopag (2.2, 4.4, and 8.8 mg/L) and grew them at 37 °C overnight. After incubation, we obtained 11 colonies that showed 2 to 4 fold increased MIC_50_ values compared to the parental strain (Spontaneous resistant mutation frequency: 5.5 × 10^−9^). Among 11 resistant colonies, 4 of them were selected either the whole genome sequencing or RT-qPCR analyses for subsequent investigations.

### 3.9. Whole-Genome Sequencing Analysis

We cultured eltrombopag-resistant *S. aureus* in MHB at 37 °C overnight and harvested cells for isolation of genomic DNA using the Wizard Genomic DNA Purification Kit (Promega, Madison, WI, USA. Genomic DNA sequences were analyzed by Illumina Miseq (CLgenomics, Chunlab, Seoul, Korea).

### 3.10. RNA Extraction and qRT-PCR

Total RNA of *S. aureus* and eltrombopag resistance strains were extracted using RNeasy Mini Kit (Qiagen, Hilden, Germany) following the manufacture’s protocol. RNA concentration was quantified by NonoVue Spectrophotometer (GE Healthcare, Chicago, USA). RT-qPCR was accomplished using the QuantiFast SYBR Green RT-PCR Kit (Qiagen, Hilden, Germany) on an ViiA7 Real-Time CRP System (Thermofisher Scientific, Waltham, MA, USA), following the program: 10 min at 50 °C, 5 min at 95 °C, followed by 40 cycles of 10 sec at 95 °C, 10 sec at 55 °C, and 20 sec at 72 °C. The relative quantification of mRNA was performed by the comparative CT (2^−ΔΔCT^) method, with the tRNA gene as an internal control. The primers designed for *tRNA* (F 5′-GATGATTGAAGGGGAAATGG-3′, R 5′-GGTGTCGCAACTTTTTCAAG-3′), *pmtA* (F 5′-ACTTAAGTGACGGTGAAGTTATC-3′, R 5′-TGCGCATGTTCTGTTAATCCT-3′), and *pmtD* (F 5′-TGGCAAATTCGATGGATAACCC-3′, R 5′-TCACGCGTAATTGTCTTAACAAC-3′) are constructed and used in this study.

### 3.11. Statistical Analysis

We performed all statistical analysis (*t* –test) using GraphPad Prism 8.0 software (GraphPad Software, San Diego, CA, USA).

## 4. Conclusions

Here, we screened 182 FDA-approved drugs to identify new therapeutic entities for stapnylococcus infections. Among 12 drugs identified with potential antibacterial activity, eltrombopag, a drug approved for thrombocytopenia, showed the most significant antibacterial activity against multidrug-resistant *S. aureus*. We confirmed that eltrombopag effectively inhibits the growth of *S. aureus* demonstrating its activity in in vitro assays of 55 clinical isolates and the cell infection model as well as in vivo assays, proposing a possible mechanism of eltrombopag-mediated bacterial growth inhibition. These results suggest the potential of eltrombopag as a repurposed drug for the treatment of multidrug resistant *S. aureus* infections, which have very limited therapeutic options. More investigations should be followed to assess potential adverse effects of eltrombopag as an antibacterial agent and to elucidate its antibacterial mechanism for further clinical development.

## Figures and Tables

**Table 1:** Summary of selected molecules with antimicrobial activity.

Compound Name	Compound Structure	% Inhibition ± SD ^a^	Class ^b^
Dronedarone HCl	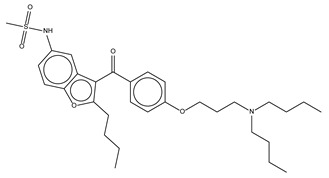	59.2 ± 0.4	Antiarrhythmic
Ceritinib	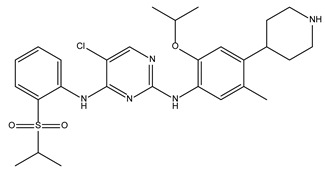	81.8 ± 2.9	Kinase inhibitor
Eltrombopag	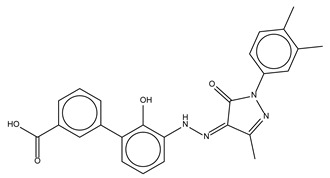	100.8 ± 0.5	Thrombopoietin receptor agonist
Ivacaftor	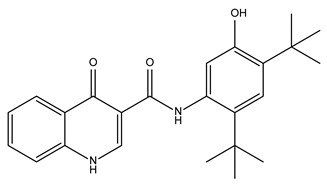	104.8 ± 0.4	Cystic fibrosis transmembrane conductance regulator (CFTR) potentiator
Tedizolid (phosphate)	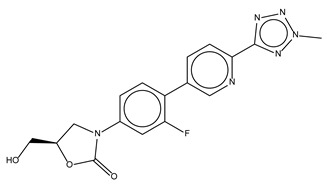	52.6 ± 2.6	Antibiotic
Fosfomycin sodium	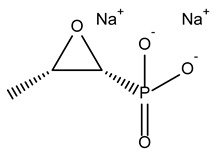	53.9 ± 0.1	Antibiotic
Rosoxacin	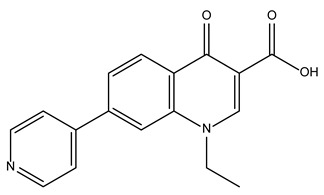	92.2 ± 0.6	Antibiotic
Fidaxomicin	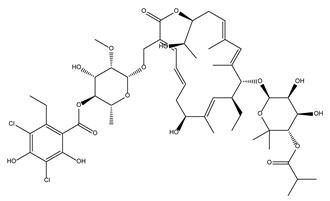	99.0 ± 0.1	Antibiotic
Retapamulin	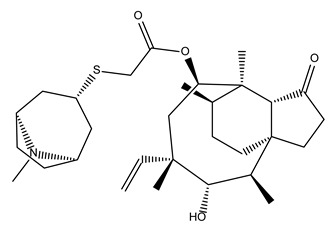	97.5 ± 1.4	Antibiotic
Cefditoren Pivoxil	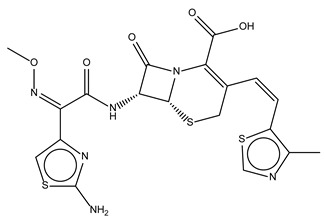	102.6 ± 0.1	Antibiotic
Ticarcillin sodium	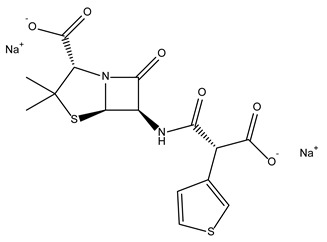	103.0 ± 1.2	Antibiotic
Cefpodoxime (free acid)	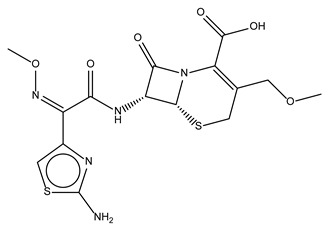	103.1 ± 0.6	Antibiotic

^a^ Bacterial growth inhibition (%) obtained from the primary screening in duplicate. ^b^ Classification of drug.

**Table 2:** Mutated gene list in eltrombopag resistant strain.

Gene ID	Gene Name	Mutation
SASA1_01682	*yedL (pmtR)*	Point mutation (P37L)
SASA1_01628	*walR*	Point mutation (E11G or D83G)
SASA1_01072	*yjbH (spxH)*	Truncation
SASA1_00966	*lytE*	Truncation
SASA1_02364	*yokF*	Truncation

## Data Availability

Not applicable.

## Supplementary Materials

The following are available online at www.mdpi.com/article/10.3390/antibiotics10111372/s1, Figure S1: *pmtA* and *pmtD* expression level (2^−ΔΔCt^) in the *S. aureus* ATCC25923 and eltrombopag resistant strains (SaEr 12, 2, 4, and 6), Table S1: Summary of antimicrobial activities of methicillin resistant *Staphylococcus aureus* (MRSA), Table S2: Summary of antimicrobial activities of Gram-positive and Gram-negative.
