# Supplementary material for: Repurposing Eltrombopag for Multidrug Resistant Staphylococcus aureus Infections
Source: Antibiotics (Basel). 2021 Nov 9;10(11):1372. doi: 10.3390/antibiotics10111372 (PMC8615030; doi:10.3390/antibiotics10111372)
Supplement: Supplementary file 1 [file antibiotics-10-01372-s001.zip › antibiotics-1446380-supplementary.pdf]

antibiotics

MDPI

Supplementary Materials

Repurposing Eltrombopag for Multidrug Resistant *Staphylococcus aureus* Infections

Hyunjung Lee [1], Jaehoan Lee [1], Juchan Hwang [1], Sinyoung Park [2], Namyoul Kim [3], Kideok Kim [3], Honggun Lee [3], David Shum [3] and Soojin Jang [1,*]

[1] Antimicrobial Resistance Laboratory, Institut Pasteur Korea, Bundang-gu, Seongnam-si, Gyeonggi-do, 13488 South Korea; hyunjung.lee@ip-korea.org (Hyunjung Lee); Jellyfocus@outlook.com (J.L.); juchan.hwang@ip-korea.org (J.H.)
[2] Animal Facility Team, Institut Pasteur Korea, Bundang-gu, Seongnam-si, Gyeonggi-do, 13488 South Korea; psy6600@daum.net
[3] Screening Discovery Platform, Institut Pasteur Korea, Bundang-gu, Seongnam-si, Gyeonggi-do, 13488 South Korea; skaduf@hanmail.net (N.K.); kideok.kim@ip-korea.org (K.K.); honggun.lee@ip-korea.org (Honggun Lee); david.shum@ip-korea.org (D.S.)
* Correspondence: soojin.jang@ip-korea.org; Tel.: +82-31-8018-8194

Table S1. Summary of antimicrobial activities of methicillin resistant *Staphylococcus aureus* (MRSA).

MDR	PEN	FOX	CEFO/Sulb	CRO/Sulb	CRO	ERY	SXT	LEV	TET
1	32	32	>128	>128	>128	>128	0.06	8	16
2	32	32	>128	>128	>128	>128	0.06	>128	32
3	16	32	>128	>128	>128	>128	0.06	16	16
4	8	32	>128	32	64	>128	0.06	0.25	0.5
5	16	32	>128	>128	>128	>128	0.06	16	1

*PEN:penicillin, FOX: cefoxitin, Sulb: sulbactam, CEFO: cefoperazone, CRO: ceftriazone, SXT: trimethoprim/sulfamethoxazole, LEV: levofloxacin, TET: tetracycline (mg/L).

Table S2. Summary of antimicrobial activities of Gram-positive and Gram-negative strains.

Strain name	Vancomycin, MIC_{50} Mean ± SD (mg/L)	Eltrombopag, MIC_{50} Mean ± SD (mg/L)	Colistin, MIC_{50} Mean ± SD (mg/L)
S. aureus ATCC25923	1.0 ± 0.05	1.5 ± 0.3	NA
S. aureus MDR1	1.0 ± 0.05	2.2 ± 0.02	NA
S. aureus MDR2	1.4 ± 0.5	2.5 ± 0.2	NA
S. aureus MDR3	1.1 ± 0.1	2.6 ± 0.02	NA
S. aureus MDR4	1.4 ± 0.5	2.5 ± 0.1	NA
S. aureus MDR5	1.4 ± 0.4	2.2 ± 0.02	NA
S. aureus MSSA1	0.5 ± 0.004	1.5 ± 0.01	NA
S. aureus MSSA2	0.5 ± 0.01	1.5 ± 0.04	NA
S. aureus MSSA3	0.5 ± 0.02	2.6 ± 0.3	NA
S. aureus MSSA4	0.5 ± 0.2	1.6 ± 0.02	NA

S. aureus MSSA5	0.5 ± 0.05	1.6 ± 0.3	NA
S. aureus MSSA6	0.5 ± 0.02	2.4 ± 0.4	NA
S. aureus MSSA7	0.4 ± 0.1	2.4 ± 0.5	NA
S. aureus MSSA8	0.8 ± 0.2	1.4 ± 0.1	NA
S. aureus MSSA9	0.4 ± 0.2	1.7 ± 0.4	NA
S. aureus MSSA10	0.5 ± 0.04	2.0 ± 0.4	NA
S. aureus MSSA11	0.5 ± 0.06	2.4 ± 0.4	NA
S. aureus MSSA12	0.6 ± 0.06	1.5 ± 0.09	NA
S. aureus MSSA13	0.5 ± 0.06	1.5 ± 0.01	NA
S. aureus MSSA14	0.8 ± 0.3	2.3 ± 0.7	NA
S. aureus MSSA15	0.5 ± 0.05	1.7 ± 0.4	NA
S. aureus MSSA16	0.5 ± 0.05	1.7 ± 0.3	NA
S. aureus MSSA17	0.6 ± 0.2	1.7 ± 0.4	NA
S. aureus MSSA18	0.5 ± 0.4	2.1 ± 0.3	NA
S. aureus MSSA19	0.6 ± 0.2	2.8 ± 0.04	NA
S. aureus MSSA20	0.5 ± 0.1	2.2 ± 0.6	NA
S. aureus MRSA1	0.4 ± 0.003	2.7 ± 0.03	NA
S. aureus MRSA2	0.4 ± 0.01	1.9 ± 0.2	NA
S. aureus MRSA3	0.4 ± 0.01	2.9 ± 0.03	NA
S. aureus MRSA4	0.3 ± 0.01	3.1 ± 1.5	NA
S. aureus MRSA5	0.4 ± 0.02	1.5 ± 0.01	NA
S. aureus MRSA6	0.5 ± 0.003	2.3 ± 0.2	NA
S. aureus MRSA7	0.3 ± 0.01	1.7 ± 0.3	NA
S. aureus MRSA8	0.7 ± 0.3	2.2 ± 0.5	NA
S. aureus MRSA9	0.6 ± 0.1	1.7 ± 0.03	NA
S. aureus MRSA10	0.6 ± 0.2	1.9 ± 0.4	NA
S. aureus MRSA11	0.6 ± 0.06	2.8 ± 0.1	NA
S. aureus MRSA12	0.9 ± 0.3	2.9 ± 1.8	NA
S. aureus MRSA13	0.5 ± 0.01	2.8 ± 0.07	NA
S. aureus MRSA14	0.6 ± 0.1	2.7 ± 0.01	NA
S. aureus MRSA15	0.4 ± 0.2	2.0 ± 0.5	NA
S. aureus MRSA16	0.8 ± 0.2	1.9 ± 0.8	NA
S. aureus MRSA17	0.7 ± 0.1	2.5 ± 0.08	NA
S. aureus MRSA18	0.5 ± 0.02	2.9 ± 0.01	NA

S. aureus MRSA19	0.4 ± 0.1	2.3 ± 0.2	NA
S. aureus MRSA20	1.0 ± 0.02	3.1 ± 1.8	NA
S. aureus MRSA21	0.5 ± 0.1	1.8 ± 0.4	NA
S. aureus MRSA22	0.3 ± 0.1	1.6 ± 0.1	NA
S. aureus MRSA23	0.5 ± 0.01	2.6 ± 0.1	NA
S. aureus MRSA24	0.6 ± 0.1	2.1 ± 0.7	NA
S. aureus MRSA25	0.6 ± 0.1	1.8 ± 0.08	NA
S. aureus MRSA26	0.6 ± 0.1	2.1 ± 0.5	NA
S. aureus MRSA27	0.6 ± 0.2	2.8 ± 0.04	NA
S. aureus MRSA28	0.6 ± 0.3	3.2 ± 1.8	NA
S. aureus MRSA29	0.7 ± 0.3	2.6 ± 0.07	NA
S. aureus MRSA30	0.8 ± 0.2	2.4 ± 0.2	NA
S. aureus eltrombopag resistant strain #2	1.4 ± 0.01	14.3 ± 1.9	NA
S. aureus eltrombopag resistant strain #4	2.6 ± 0.8	7.5 ± 3.0	NA
S. aureus eltrombopag resistant strain #6	1.4 ± 0.02	16.2 ± 2.8	NA
S. pneumoniae 49619	0.3 ± 0.06	0.2 ± 0.001	NA
S. pneumoniae 700904	0.3 ± 0.02	0.3 ± 0.08	NA
P. aeruginosa PAO1	NA	No activity	4.9 ± 0.07
A. baumannii	NA	No activity	3.0 ± 0.6
K. pneumoniae	NA	No activity	12.3 ± 1.6

*NA: Not Available.

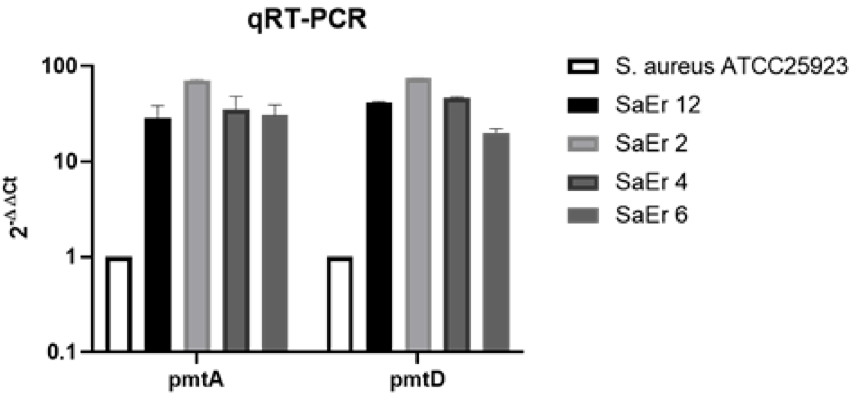

Figure S1. *pmtA* and *pmtD* expression level ($2^{-\Delta\Delta Ct}$) in the *S. aureus* ATCC25923 and eltrombopag resistant strains (SaEr 12, 2, 4, and 6).
